# Wearable Fall Detection System with Real-Time Localization and Notification Capabilities

**DOI:** 10.3390/s25123632

**Published:** 2025-06-10

**Authors:** Chin-Kun Tseng, Shi-Jia Huang, Lih-Jen Kau

**Affiliations:** 1 Department of Electronic Engineering, National Taipei University of Technology, Taipei 106344, Taiwan; tsengchinkun@gmail.com (C.-K.T.); taless9273511@gmail.com (S.-J.H.); 2 Tri-Service General Hospital Songshan Branch, Taipei 105309, Taiwan; 3 National Defense Medical Center, Taipei 114201, Taiwan

**Keywords:** fall detection, inertial measurement unit (IMU), Global Positioning System (GPS), Narrowband Internet of Things (NB-IoT)

## Abstract

Despite significant progress in fall detection systems, many of the proposed algorithms remain difficult to implement in real-world applications. A common limitation is the lack of location awareness, especially in outdoor scenarios where accurately determining the fall location is crucial for a timely emergency response. Moreover, the complexity of many existing algorithms poses a challenge for deployment on edge devices, such as wearable systems, which are constrained by limited computational resources and battery life. As a result, these solutions are often impractical for long-term, continuous use in practical settings. To address the aforementioned issues, we developed a portable, wearable device that integrates a microcontroller (MCU), an inertial sensor, and a chip module featuring Global Positioning System (GPS) and Narrowband Internet of Things (NB-IoT) technologies. A low-complexity algorithm based on a finite-state machine was employed to detect fall events, enabling the module to meet the requirements for long-term outdoor use. The proposed algorithm is capable of filtering out eight types of daily activities—running, walking, sitting, ascending stairs, descending stairs, stepping, jumping, and rapid sitting—while detecting four types of falls: forward, backward, left, and right. In case a fall event is detected, the device immediately transmits a fall alert and GPS coordinates to a designated server via NB-IoT. The server then forwards the alert to a specified communication application. Experimental tests demonstrated the system’s effectiveness in outdoor environments. A total of 6750 samples were collected from fifteen test participants, including 6000 daily activity samples and 750 fall events. The system achieved an average sensitivity of 97.9%, an average specificity of 99.9%, and an overall accuracy of 99.7%. The implementation of this system provides enhanced safety assurance for elderly individuals during outdoor activities.

## 1. Introduction

Due to the continuous rise in the proportion of elderly populations, many countries around the world are currently facing a significant demographic shift. In the context of rapid societal aging, the shortage of care-giving personnel has become increasingly severe. Ensuring the safety and care of the elderly has become an important issue that is receiving growing attention. In Taiwan, according to the population projection report by the National Development Council, Taiwan entered an “aging society” at the end of March 2018. This year (2025), it will become a “super-aged society”, meaning that the elderly population aged 65 and above will account for more than 20% of the total national population. It is estimated that by 2070, the proportion of elderly people in Taiwan will increase significantly to 43.6% [1].

Statistics show that among the various causes of death among the elderly—aside from various diseases—accidents and injuries are also one of the top ten leading causes of death among the elderly. Among these accidents, falls rank second, just after traffic accidents, as the leading cause of injury-related deaths in individuals aged 65 and above. Falls not only pose a serious threat to the health of the elderly, but severe falls also carry the risk of long-term bedridden conditions. This not only imposes a heavy burden on family caregivers and finances but also significantly impacts the healthcare system’s required resources and has a negative impact on the socioeconomic structure.

To prevent falls among the elderly or quickly detect fall incidents as they occur, many studies have focused on designing assistive living systems for seniors and developing fall detection devices and detection algorithms. Existing fall detection devices and algorithms can be broadly classified into two main types. One type involves fall detection through wearable devices, while the other type involves deploying sensors in specific environments, such as the living room or lounge, to detect fall incidents [2].

In the field of wearable devices, most studies integrate inertial sensors or pressure sensors to implement dedicated fall detection modules. For example, the authors of [3] used an accelerometer and applied the K-Nearest Neighbor (KNN) classifier, as well as a support vector machine (SVM) with linear, quadratic, and polynomial kernels, to detect fall events. In [4,5], a module consisting of an accelerometer and a gyroscope was positioned at the waist of elderly individuals for fall detection, and a thresholding method was applied based on the values from both sensors. In [6], an accelerometer, gyroscope, and pressure sensor were used, followed by the application of a feedforward neural network (FFNN) to detect fall events. In addition to dedicated modules, since most current smartphones are equipped with inertial sensors, such as accelerometers and gyroscopes, and have excellent computational capabilities, some research has directly utilized the built-in inertial sensors of smartphones in combination with apps running on these devices for user behavior and fall event detection [7]. Furthermore, the authors of [8] installed accelerometers and gyroscopes on the frame of smart glasses and employed a complementary filter to eliminate the angle measurement instability and drift caused by these sensors. Finally, a series of threshold tests were used to determine whether the user had experienced a fall event [8]. In [9], a wearable module consisting of accelerometer and gyroscope sensors for fall detection was proposed, and a fuzzy logic-based algorithm was then employed as a web service for real-time detection, ensuring high accuracy and low false alarms. However, the large amount of sensor data needed to be transmitted to the cloud via the network, which clearly resulted in significant bandwidth burden and power consumption. In [10], accelerometers and gyroscopes were also used for fall event detection, and a convolutional neural network was employed to classify fall events. Additionally, the study analyzed whether incorporating angular velocity data improved classification performance and found that gyroscope data could enhance detection, depending on the classifier and feature set used.

In the category of deploying sensors in elderly living environments, various contactless sensing technologies are used for fall detection, including radar technologies (such as IR-UWB and millimeter-wave radar) and WiFi signals. Some systems rely on radar or WiFi sensing for real-time fall detection, while others use cameras combined with pose estimation and artificial intelligence techniques to enhance detection accuracy. In [11], a three-stage fall detection method utilizing an impulse radio ultra-wideband (IR-UWB) radar was proposed, which included signal pre-processing, feature extraction, and fall classification using a decision threshold [11]. Another study employed radar sensors combined with time–frequency analysis and convolutional neural networks (CNNs) to classify fall events, achieving high accuracy in a contactless manner [12]. A millimeter-wave radar-based system was introduced, integrating neural networks and information fusion techniques for improved fall detection [13]. A pose estimation-based methodology for fall detection was developed, utilizing camera data and artificial intelligence edge computing for real-time processing [14]. A real-time contactless fall detection system using commodity WiFi devices and machine learning algorithms was proposed to distinguish between falls and normal activities [15]. Lastly, a WiFi-based fall detection system using recurrent neural networks (RNNs) effectively modeled temporal patterns in WiFi signal data to detect falls in smart home environments [16].

By reviewing relevant studies, it can be found that deploying sensors in the environment is not only limited by the detection location, making it difficult to apply in outdoor settings, but, also, the construction cost is usually high. Additionally, using imaging methods for fall detection raises concerns about privacy invasion. On the other hand, using radar in various frequency bands for fall detection can avoid privacy concerns; however, radar waves are highly sensitive and more susceptible to interference, leading to false positive misjudgments [2].

In contrast, when using wearable devices for fall detection, the use of smart glasses may cause discomfort due to wearing habits, which may affect elderly individuals’ willingness to adopt them. Although smartphones are widely available and typically have positioning and messaging capabilities, elderly individuals are usually less familiar with operating smartphones. The way they hold and use the device can also increase the difficulty for algorithms to make accurate judgments. Upon reviewing various studies that use homemade wearable devices for fall detection, we also noticed that most of these studies focus only on fall detection capabilities without providing positioning or event notification functions. As a result, it is difficult to achieve timely rescue during a fall incident.

In addition to using wearable devices or environmental deployments for sensing, a considerable number of fall detection algorithms have been proposed in recent years. Particularly with the rapid advancement of machine learning and artificial intelligence, the integration of related algorithms with various sensor modalities has not only improved the accuracy of fall detection but has also been successfully applied to various activity recognition tasks, thereby creating more possibilities for practical applications. In [17], a vision-based fall detection method was proposed using a single Kinect sensor to capture skeletal motion data. The algorithm employed a hybrid architecture that combined 3D convolutional neural networks (3D-CNNs) and Long Short-Term Memory (LSTM) networks to extract spatiotemporal features for fall classification. The dataset used in this study was collected from 10 participants and included several types of falls (e.g., forward, backward, and lateral) along with multiple daily activities. Performance evaluation based on accuracy, sensitivity, and F1-score demonstrated high detection effectiveness. In [18], the authors presented a fall detection approach based on a single tri-axial accelerometer and a convolutional neural network (CNN). The algorithm was developed using TensorFlow and Keras, and exclusively relied on two publicly available datasets, MobiAct and SisFall, without any additional data collection. It was tested on various types of falls (forward, backward, left, and right) and daily activities such as walking, sitting, running, and stair use. The method achieved up to 98.98% sensitivity and 99.63% specificity, as evaluated using metrics including accuracy, sensitivity, specificity, and precision.

A deep learning-based activity recognition framework was proposed in [19], employing the Deep ConvLSTM model enhanced with a self-attention mechanism. This approach leveraged data from multiple wearable sensors and was implemented using TensorFlow and Keras. Training and testing were conducted using several public datasets, including MHEALTH, OPPORTUNITY, Skoda, UCI HAR, WISDM, and PAMAP2, without additional data collection. The study primarily focused on a broad range of daily activities, such as walking, running, sitting, cycling, and stair climbing, rather than fall-specific detection. Nonetheless, the model achieved consistently high performance across datasets, as measured by the accuracy, precision, sensitivity, and specificity. In [20], a radar-based fall detection system was introduced, incorporating multiple ultra-wideband (UWB) radars and a deep neural network composed of CNN and LSTM layers. The system was implemented on a desktop platform using TensorFlow, and data were collected in a realistic 40 m² apartment environment. Ten participants performed simulated falls under four different scenarios, although daily activities were not evaluated. The model achieved an accuracy of approximately 90%, demonstrating the potential of UWB radar combined with deep learning for robust fall detection.

A novel sensor-based activity recognition method was proposed in [21], utilizing a channel attention mechanism in the discrete cosine transform (DCT) domain to enhance frequency feature embedding. The model used data from multiple inertial measurement units (IMUs) and was implemented using TensorFlow. Evaluation was performed on several public datasets, including UCI HAR, WISDM, PAMAP2, and OPPORTUNITY. Although the study did not specifically address fall detection, it achieved approximately 98% accuracy in recognizing various daily activities such as walking, running, sitting, and stair climbing. This result highlights the effectiveness of frequency–domain feature enhancement in sensor-based activity recognition. Furthermore, a video-based fall detection approach using a constrained generative adversarial network (cGAN) that operated on human pose representations extracted from RGB video was presented in [22]. The system used a single RGB camera to capture pose sequences, predicting future frames and computing anomaly scores to detect falls. The model was trained and evaluated on public datasets, including the UR Fall Detection Dataset and the Le2i Fall Detection Dataset. Although specific fall directions and daily activities were not separately analyzed, the method achieved a high accuracy of 99.61%, demonstrating strong performance in unsupervised fall detection based on human pose dynamics.

While multimodal fusion and deep learning algorithms have demonstrated notable improvements in fall detection accuracy, their practical implementation on wearable devices based on microcontrollers presents significant challenges. These algorithms typically require substantial computational resources, e.g., memory, and power, which exceed the capabilities of most low-power embedded systems commonly used in wearables. Microcontroller-based devices, designed for energy efficiency and compact form factors, are inherently limited in processing power and battery life. Consequently, the integration of complex, multimodal data fusion and deep learning inference can lead to rapid power consumption and thermal constraints, reducing the usability and operational lifespan of the device. Therefore, despite their advantages in performance, such approaches are often impractical for wearable systems intended for continuous, long-term monitoring in real-world settings.

In this manuscript, we propose a wearable fall detection device that integrates an inertial measurement unit (IMU), comprising an accelerometer and gyroscope, with a low-complexity finite state machine (FSM) algorithm. The FSM sequentially evaluates fall-related features and terminates further processing as soon as a condition fails, similar to a cascade classifier. This structure significantly reduces computational load and power consumption, making it well-suited for microcontrollers with limited processing capacity. The device is built around a low-power microcontroller capable of real-time execution, ensuring long-term and continuous operation. To enhance usability for elderly individuals during outdoor activities, the module also incorporates a lithium-ion battery and a power management chip and is worn around the waist. In the event of a fall, the integrated Global Navigation Satellite System (GNSS, and, specifically, GPS in this manuscript) module acquires the fall location, and the NB-IoT module transmits the coordinates to a cloud server. This information is then forwarded via instant messaging to pre-designated family members or emergency responders, enabling timely assistance. With the aforementioned functionality, the proposed system provides a practical and reliable solution for monitoring the safety of the elderly.

Overall, the main contributions of this work are as follows:

- A low-complexity FSM-based algorithm is implemented on a microcontroller without relying on any external computing resources.
- The FSM employs a staged decision-making process, similar to a cascade classifier. If any stage fails to meet the defined criteria, the possibility of a fall is immediately excluded, thus avoiding subsequent evaluations and reducing computational load and power consumption.
- The fall detection module integrates both GPS and NB-IoT communication, enabling real-time location tracking and notification at the moment of a fall.
- Real-time notification is supported through integration with instant messaging applications. The system also incorporates the Google Maps API to relay the incident location to family members or designated emergency contacts, thereby improving outdoor safety for elderly users.
- The system achieves low-power operation. Even with all features, i.e., GPS and NB-IoT, continuously active, the battery retains at least 64% of its capacity after 10 h of use, ensuring full-day operational reliability for outdoor activities. When the SIM7000G module is configured in Normal Idle mode, i.e., with GNSS disabled (wake-up on demand) and NB-IoT in the RRC Idle state, the average current consumption of the module is approximately 20 mA. Based on our endurance tests, with the selected 2000 mAh lithium-ion battery, the system can operate for up to 4.2 days. Furthermore, if the MCU is also set to low-power mode and the IMU’s “Wake-on-Shake” function is used to trigger wake-ups, the estimated operational time can be extended to approximately 16.7 days.

The rest of this paper is organized as follows: Section 2 introduces the proposed system architecture, outlining the components of the system. IMU signal acquisition and analysis are presented in Section 3. In addition, this section provides a detailed discussion of our observations on the differences in IMU signal patterns between falls and daily activities. Based on these observations, Section 4 presents the proposed finite state machine-based fall detection method in detail. The experimental results of the proposed approach and comparisons with existing state-of-the-art fall detection algorithms are provided in Section 5. The system limitations and potential research directions, particularly the development of next-generation wearable fall detection devices that integrate AI technologies for algorithm optimization and personalized configuration, are given in Section 6. Finally, Section 7 concludes this paper.

## 2. Proposed System Architecture

**Figure 1:**
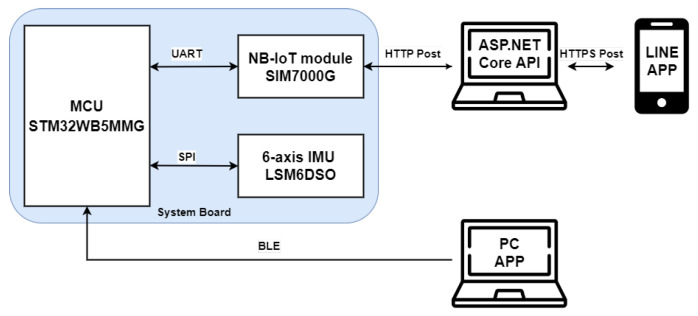
Proposed fall event detection and message delivery architecture.

The proposed system architecture is shown in Figure 1. As can be seen in Figure 1, the proposed fall detection system is composed of three major parts, one of which is the main module worn by the elderly, another is the server built in the cloud, and the third is a laptop with an offline application program (APP) for data analysis and algorithm development. The wearable main module can be further divided into three parts: the microcontroller that is responsible for algorithm computation, the six-axis inertial sensor (including a three-axis accelerometer and a three-axis gyroscope) responsible for posture sensing, and the SIM7000G module, which is composed of a GPS receiver and an NB-IoT module responsible for elderly positioning and rescue message transmission. As for the cloud server, it can be further divided into two parts: the ASP.NET Core API and the LINE messaging software.

During the algorithm development phase, the microcontroller read posture data from the six-axis inertial sensor through the SPI interface. This data was then transmitted to the laptop with the offline analysis application program for data analysis via the Bluetooth Low Energy (BLE) protocol. Please note that the laptop with the offline application program (APP) was used only for data analysis during the algorithm development phase and will not be used once the algorithm is finalized. During the system’s operation, the microcontroller continued to read posture data from the six-axis inertial sensor via the SPI interface and performed fall event detection based on the algorithm. Once a fall event was confirmed, the microcontroller communicated with the SIM7000G module via the UART interface. The SIM7000G module then obtained the GPS coordinates of the elderly and transmitted the GPS coordinates and fall alert to the cloud server with the NB-IoT module. The server subsequently pushed the message to the LINE application to promptly notify family members or caregivers.

### 2.1. Proposed Wearable Module

**Figure 2:**
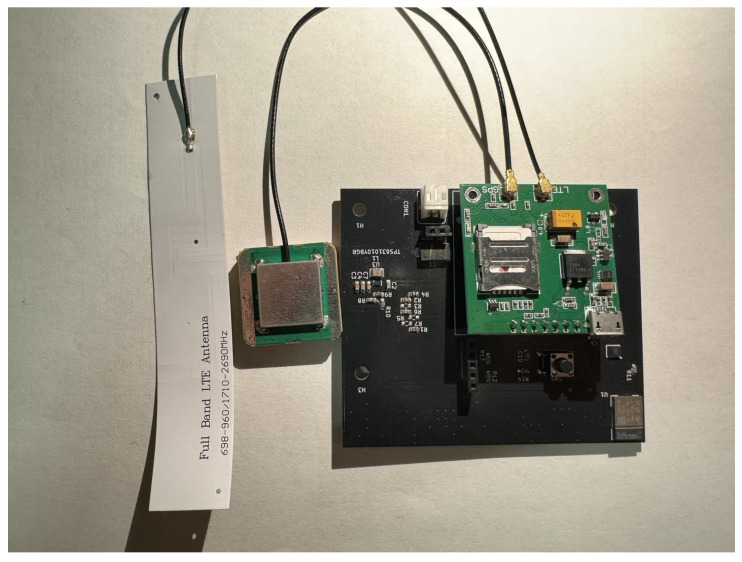
The proposed wearable module.

**Figure 3:**
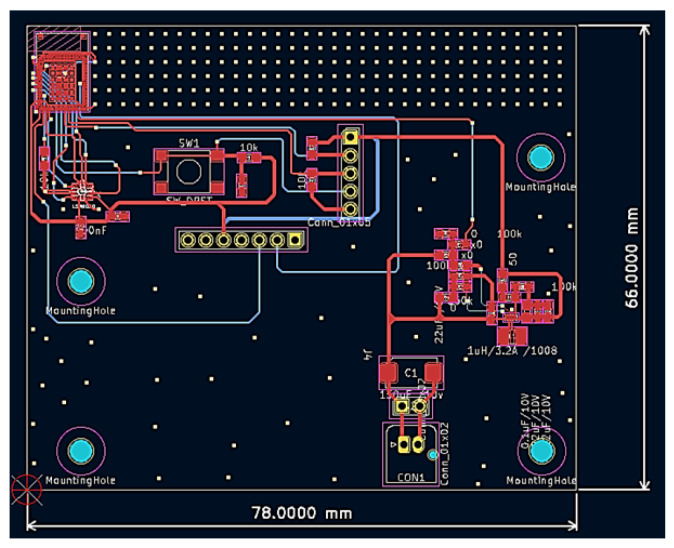
PCB layout of the system board.

**Figure 4:**
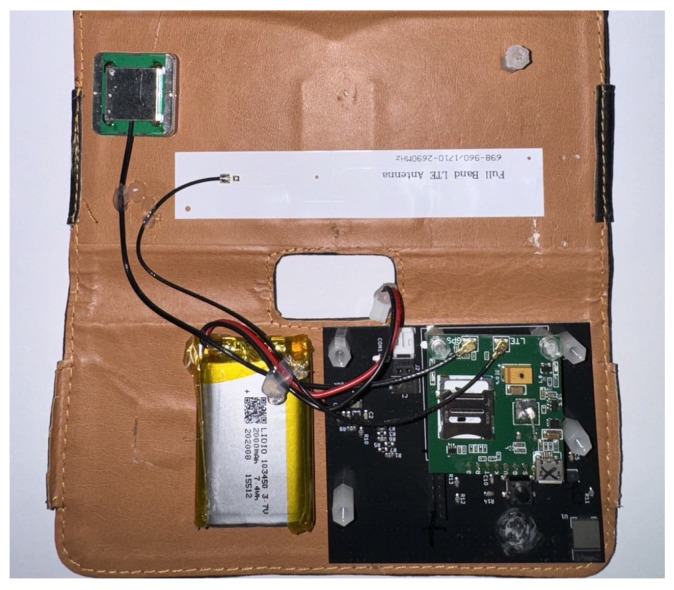
Internal component configuration of the fall detection module.

The core of this study, namely, the wearable main module, is shown in Figure 2. As mentioned above, the main module consists of three functional components, i.e., a microcontroller, a six-axis inertial sensor, and an NB-IoT module. Physically, the main module is primarily made up of two stacked printed circuit boards (PCBs), the lower black system board and the upper green SIM7000G module. The microcontroller, responsible for computation, and the six-axis inertial sensor, responsible for posture detection, are placed on the system board. Additionally, the system board also includes a power management chip, which regulates the voltage of the lithium-ion battery and provides power to the module. The PCB layout of the system board is shown in Figure 3. In addition, the green printed circuit board (PCB) stacked above the black system board is the SIM7000G module. Since the SIM7000G module integrates both GPS positioning and NB-IoT communication functions, a GPS receiver and a mobile communication antenna are also required. The smaller green square module in Figure 2 is the GPS receiver, while the white elongated object on the left is the full-band ution (LTE) antenna. We integrated a system board containing the MCU and IMU, the SIM7000G module, a 2000 mAh lithium-ion battery, the GPS receiver, and the LTE antenna into a wearable pouch case, similar to an eyeglass case, as shown in Figure 4. Once the integration was completed, the pouch case could be worn by elderly individuals for fall detection. Below, we will introduce the specifications of the main components of this module.

#### 2.1.1. Processing Unit: STM32WB5MMG

The core of the fall detection module was based on the STM32WB5MMG microcontroller. The STM32WB5MMG is a high-performance microcontroller that supports the Bluetooth Low Energy (BLE) wireless communication protocol. It integrates a Cortex-M0+ chip for radio frequency transceiver operations and a Cortex-M4 chip as the main processing core. The package size is 7.3 mm × 11 mm and the operating temperature range is −40 to 85 °C [23].

This MCU reads posture data from the six-axis inertial sensor via the SPI interface. During the algorithm development phase, the data from the inertial sensor was transmitted via BLE to an offline analysis application program for data processing. Once the algorithm development is finalized and the module is in actual operation, the STM32WB5MMG microcontroller will continuously read data from the inertial sensor and use a finite state machine to determine whether a fall event has occurred. If a fall event is confirmed, the microcontroller will communicate with the SIM7000G module via a serial UART interface. After that, the fall event and the GPS coordinates of the elderly person’s location will be sent to the cloud API server via the NB-IoT functionality of the SIM7000G module.

#### 2.1.2. Six-Axis Inertial Measurement Unit

In this study, the inertial measurement unit (IMU) LSM6DSO was selected to acquire the posture of the elderly. The LSM6DSO is a high-performance, six-axis inertial measurement unit (IMU) produced by STMicroelectronics. It integrates a three-axis accelerometer and a three-axis gyroscope, designed for low power consumption and high-performance applications. It is highly suitable for scenarios such as the Internet of Things (IoT) and wearable devices. The technical specifications of the LSM6DSO are summarized as follows [24].

- Acceleration range: ±2 g/±4 g/±8 g/±16 g
- Angular velocity range: ±125 dps/±250 dps/±500 dps/±1000 dps/±2000 dps
- Package size: 2.5 × 3 × 0.83 mm

In the experimental analysis, setting the acceleration range to ±8 g and the angular velocity range to ±500 dps was sufficient to meet the fall detection requirements. Therefore, this configuration was chosen for the experiments and analysis in this study.

#### 2.1.3. The SIM7000G Module

The SIM7000G is a multifunctional wireless module produced by SIMCom Wireless Solutions Limited, supporting LTE CAT-M1, NB-IoT, and GPRS/EDGE communication protocols, with built-in GNSS and GPS positioning capabilities. In this study, when a fall event was detected, the system acquired GPS positioning information and transmitted the fall alert along with the GPS data to the cloud API server via NB-IoT and then the server sent the alert information to the LINE messaging application to enable real-time notifications and location-based rescue functionality.

### 2.2. Cloud Server

The Cloud Server designed in this study included two main functional modules: one was the “ASP.NET Core API” and the other was the function of “LINE API”. A brief description follows.

#### 2.2.1. ASP.NET Core API

ASP.NET Core API is a powerful, open-source, and cross-platform framework developed by Microsoft for building network applications, specifically designed for API service development. ASP.NET Core provides a high-performance, scalable, and modular construction approach. In this study, the API server was responsible for handling fall alerts and GPS positioning information from the NB-IoT module. After these data were transmitted to the server via the API, the server forwarded the real-time notifications to the LINE APP, enabling a quick response and location tracking for fall events.

#### 2.2.2. LINE API

**Figure 5:**
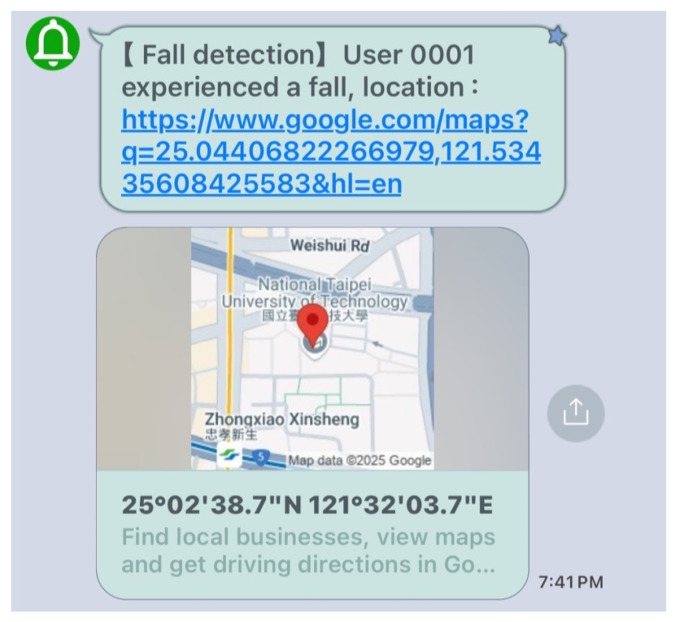
LINE notification when an elderly person experiences a fall event.

LINE is an instant messaging application developed and launched in Japan by LINE Corporation, a subsidiary of South Korea’s Naver Corporation. Since its launch in 2011, it has become a highly popular messaging APP in Asia, especially in countries like Japan, Taiwan, Thailand, and Indonesia. In addition to offering basic messaging and calling features, LINE provides a wide range of services, making it a versatile social platform. In this study, we integrated the LINE API to incorporate the message push service into the LINE platform, leveraging this feature to send GPS location information and fall alert notifications. An illustration of the LINE notification message triggered when an elderly person experiences a fall is shown in Figure 5.

### 2.3. Offline Analysis APP

**Figure 6:**
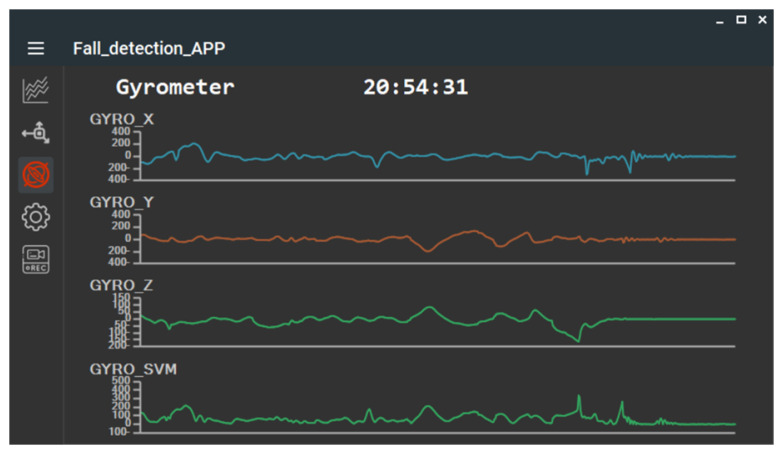
The waveform of accelerometer data for the *X*, *Y*, and *Z* axes displayed on the offline APP interface.

**Figure 7:**
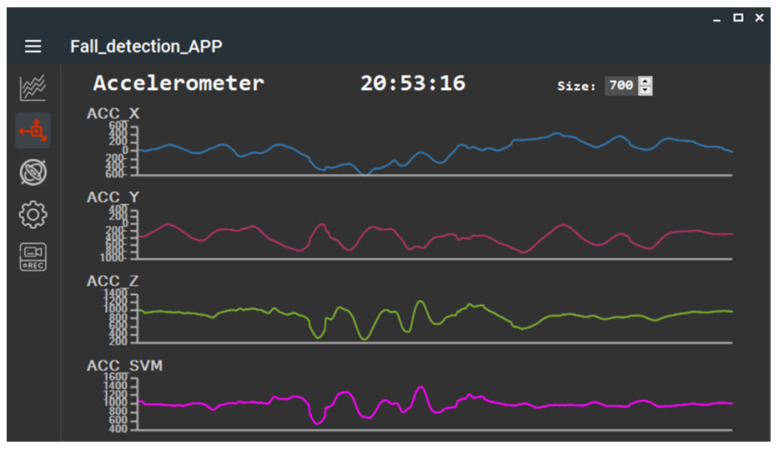
The waveform of gyroscope data for the *X*, *Y*, and *Z* axes displayed on the offline APP interface.

**Figure 8:**
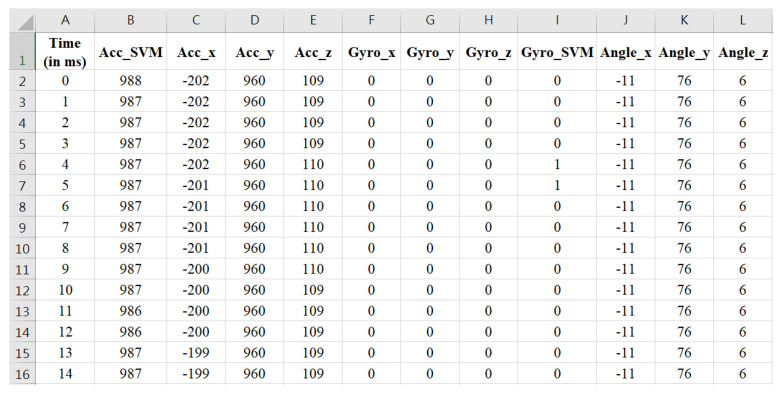
The CSV format of accelerometer and gyroscope data exported by the offline APP.

During the algorithm development phase, the STM32WB5MMG MCU transmitted the collected IMU data via BLE to a laptop running an offline application for analysis. In this study, the Offline Analysis APP was developed using a C# WinForm application, which received accelerometer and gyroscope data from the main module. To facilitate algorithm analysis, the APP’s interface also displayed waveform variations for the accelerometer and gyroscope sensors along each individual axis, as shown in Figure 6 and Figure 7. Additionally, the IMU data collected by the Offline Analysis APP was simultaneously saved in CSV format, as illustrated in Figure 8.

## 3. Signal Acquisition and Analysis

In this section, we first introduce the wearing method of the proposed fall detection module. We then analyze eight types of daily activities, including running, walking, stepping, going upstairs, going downstairs, jumping, sitting, and quick sitting, as well as four kinds of fall postures, including forward fall, rightward fall, leftward fall, and backward fall. The variations in the IMU signals and our observations are discussed. Finally, based on the aforementioned analysis, we systematically propose a set of effective features that can be derived from the IMU device for distinguishing fall events from daily activities.

### 3.1. Sensor Placement

**Figure 9:**
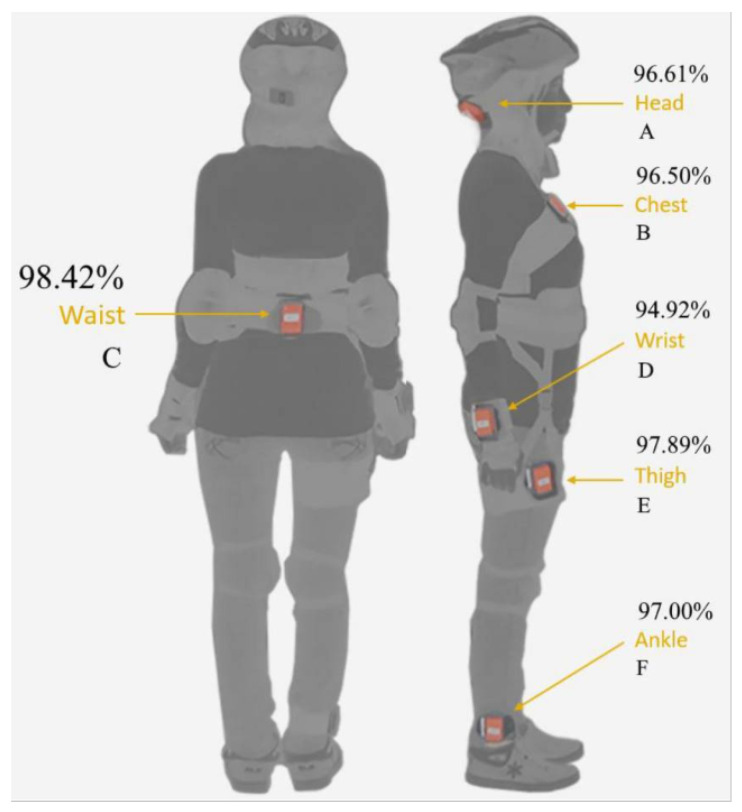
Detection accuracy vs. sensor placement [25].

**Figure 10:**
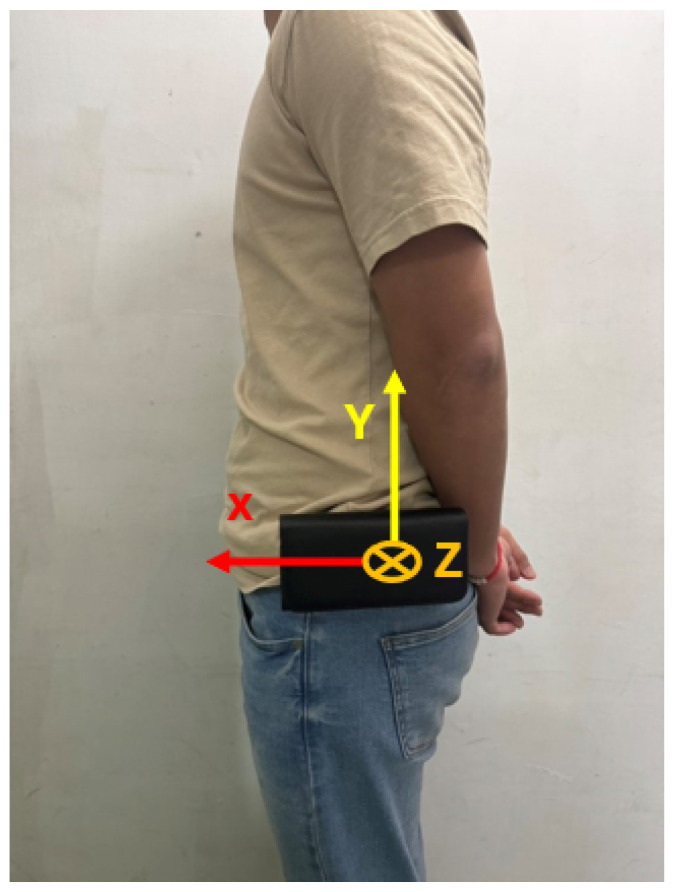
Schematic of the fall detection module worn on the waist.

To investigate the impact of sensor wearing methods and placement on fall detection performance, a detailed analysis and comparison were presented in [25]. The study tested sensor placements on the right wrist, right thigh, right ankle, chest, waist, and head. A total of 14 participants were invited to take part in the experiments, where they performed 20 simulated falls and 16 activities of daily living. The performance results of sensor placement at different positions are shown in Figure 9. It was found that placing the sensor on the waist, which achieved an accuracy of up to 98.42%, was the most ideal location. Based on the analysis results in [25], the proposed fall detection module was placed on the user’s waist in this study, as shown in Figure 10.

### 3.2. Signal Acquisition

Most wearable fall detection algorithms rely on inertial measurement units (IMUs) to assess the user’s posture and activity. However, due to variations in wearing positions and methods, as well as the numerous potential directions of fall postures, the complexity of judgment increases. To simplify this, we first calculated the sum of the squared components of the three-axis accelerometer and gyroscope signals along the (*x*, *y*, *z*) axes and then took the square root as in (1) and (2). This process reduced the original three-dimensional signals into the so-called one-dimensional signal vector magnitude (SVM) [4,14].

(1) $$A_{SVM}=\sqrt{a_{x}^{2}+a_{y}^{2}+a_{z}^{2}},$$

where $A_{SVM}$ is the dimension-reduced signal vector magnitude of the accelerometer and $a_{x}$, $a_{y}$, and $a_{z}$ are the acceleration components along the *X*-axis, *Y*-axis, and *Z*-axis, respectively.

(2) $$G_{SVM}=\sqrt{g_{x}^{2}+g_{y}^{2}+g_{z}^{2}},$$

where $G_{SVM}$ is the dimension-reduced signal vector magnitude of the gyroscope and $g_{x}$, $g_{y}$, and $g_{z}$ are the angular velocity components along the *X*-axis, *Y*-axis, and *Z*-axis, respectively.

In the proposed system, we used a sampling frequency of 100Hz to read data from the three-axis accelerometer and the three-axis gyroscope. During the data acquisition process, the data was simultaneously output by the IMU to ensure timestamp alignment. The acceleration signal, $A_{SVM}$, and the gyroscope signal, $G_{SVM}$, were then calculated using (1) and (2), respectively.

### 3.3. Fall and Daily Activity Patterns

**Figure 11:**
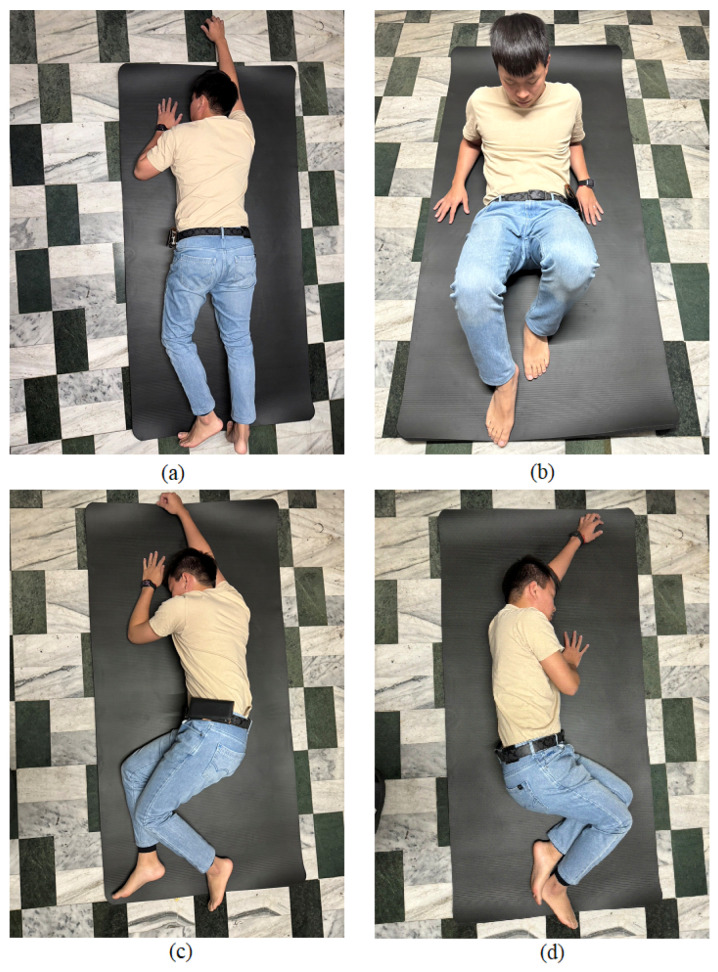
Scenario of four types of falls. (**a**) Forward fall (**b**) Backward fall (**c**) Right-side fall (**d**) Left-side fall.

**Figure 12:**
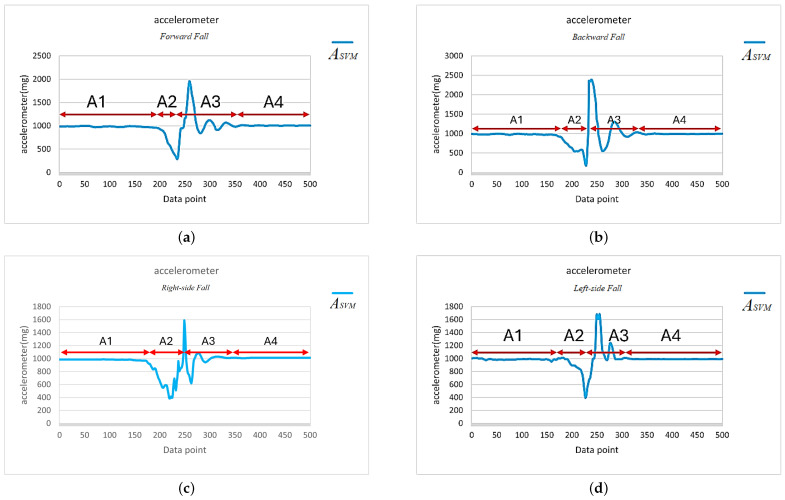
SVM signals of the accelerometer for four different fall directions. (**a**) Forward fall. (**b**) Backward fall. (**c**) Right-side fall. (**d**) Left-side fall.

**Figure 13:**
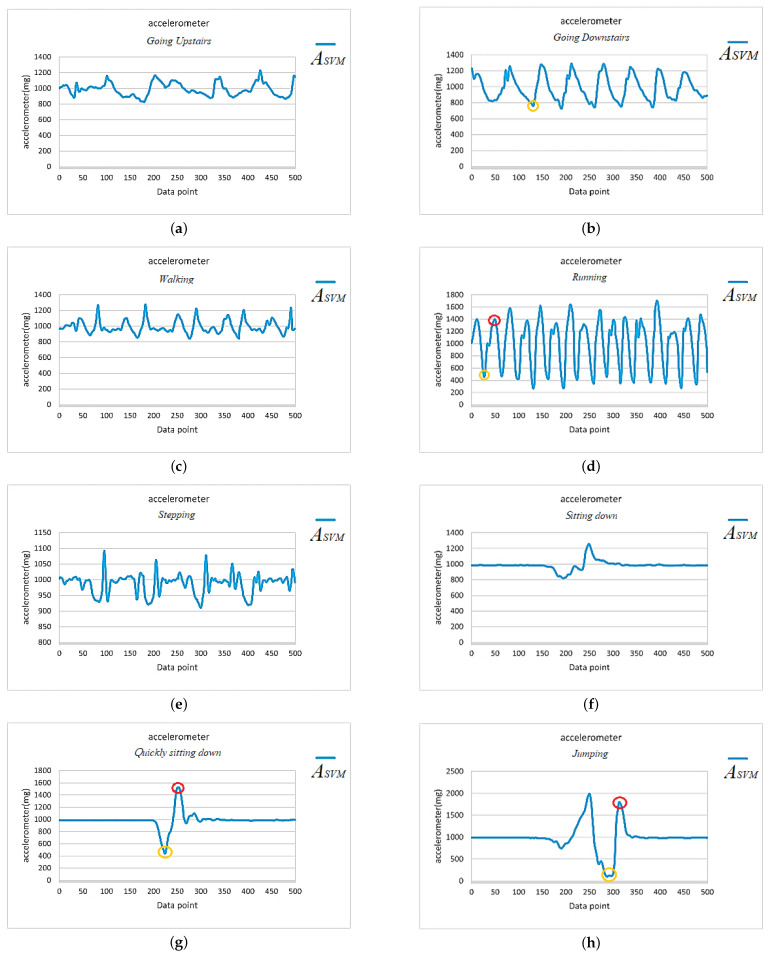
SVM signals of the accelerometer for eight different daily activities. The orange circles indicate instances where $A_{SVM}$ is below 0.8 G and the red circles indicate when it exceeds 1.4 G. (**a**) Going upstairs. (**b**) Going downstairs. (**c**) Walking. (**d**) Running. (**e**) Stepping. (**f**) Sitting down. (**g**) Quickly sitting down. (**h**) Jumping.

In this paper, we tested four different fall directions, including forward fall, right-side fall, left-side fall, and backward fall, as in Figure 11. During the algorithm development and testing period, the subjects randomly simulated falls at different speeds, including what is known as a limp fall or heavy fall. Additionally, to avoid misjudgments by the algorithm due to daily activities, we also analyzed and tested the effects of eight kinds of daily activities, including running, walking, sitting down, going upstairs, going downstairs, stepping, jumping, and quickly sitting down, on the IMU and algorithm. The signal vector magnitude of the accelerometer for the four types of fall directions and eight daily activities are shown in Figure 12 and Figure 13, respectively.

To facilitate signal observation and analysis, during the algorithm development and analysis phase, we categorized the four kinds of fall scenarios and eight daily activities into two main action types: continuous activities and single actions, as shown below.

- Continuous activity types: including running, walking, stepping, going upstairs, and going downstairs.
- Single action types: including jumping, sitting down, quickly sitting down, and falls in the forward, right-side, left-side, and backward directions.

Continuous activity types continued after initiation, with the system recording continuously for 5 s, obtaining 500 data points, and calculating the signal vector magnitude of the accelerometer, as shown in Figure 13a–e. The reason for recording continuously for 5 s was that the duration of a typical fall, from occurrence to impact, lasts about 2 to 3 s. Therefore, a 5-second recording period allowed for the observation of the complete fall waveform. For single action types, during the testing process, each action started from a stationary state, with only one action performed, followed by the calculation of the signal vector magnitude of the accelerometer, as shown in Figure 12a–d and Figure 13f–h.

### 3.4. Observations

**Figure 14:**
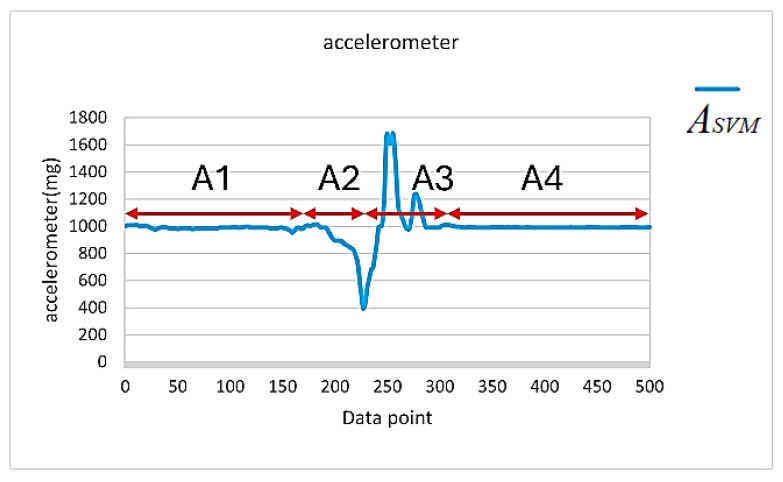
Fall event stages, using a left-side fall as an example.

Through a detailed analysis of the signal vector magnitude waveform corresponding to the accelerometer during a fall event, we could categorize the fall event into four distinct stages, as shown in Figure 14 (an example of a left-side fall). The four stages observed are described below.

1. Static standing (Stage A1): The signal vector magnitude of the accelerometer showed minimal variation, displaying a flat and stable curve with no significant motion occurring.
2. Loss of balance (Stage A2): When a fall occurred, the human torso experienced an unbalanced state, causing fluctuations in the signal vector magnitude. When the subject was unable to regain balance and began to fall, they entered a brief period of weightlessness. During this time, the accelerometer’s signal vector magnitude dropped below 1 G and continued to decrease. Based on experimental observations and statistics, during a fall event, the signal vector magnitude of the accelerometer will drop below 0.8 G during this stage.
3. Impact with the ground (Stage A3): The fall process was followed by a rapid increase in the signal vector magnitude due to the impact with the ground, which then quickly dropped until the subject’s movement in the vertical direction to the ground stopped. Based on experimental statistical analysis, at the moment of impact with the ground, the signal vector magnitude exceeded 1.4 G, indicating that the subject experienced a large force in the direction opposite to their motion at the moment of impact.
4. Stable recovery (Stage A4): After the impact with the ground, the subject either fell onto their back or sat on the ground. Since there were no significant movement changes in the subject’s posture during this period, the accelerometer’s signal vector magnitude entered the recovery phase. The signal gradually stabilized and returned to a level close to the static 1 G.

By observing the $A_{SVM}$ waveform in Figure 14, we can see that the total number of data points in the A2 and A3 stages did not exceed 200 points (i.e., 2 s). Therefore, a value of $A_{SVM}$ less than 0.8 G was used as the trigger point in this study. In other words, once $A_{SVM}$ dropped below 0.8 G, the microcontroller began recording 200 data points and checked if any of the recorded points had an $A_{SVM}$ value exceeding 1.4 G. If an $A_{SVM}$ value greater than 1.4 G was detected, this meant that a fall event may have occurred, but a further assessment was required.

We further examined the $A_{SVM}$ signals of the eight daily activities in Figure 13 and found that both going upstairs and going downstairs satisfied the condition for the trigger point, i.e., $A_{SVM}$ was less than or equal to 0.8 G, as indicated by the orange circles in Figure 13a,b. Moreover, activities such as running, quickly sitting down, and jumping not only met the condition for trigger point activation but also caused the $A_{SVM}$ signal to exceed 1.4 G within the 200 recorded data points after activation, as indicated by the red circles in Figure 13d,g,h. Clearly, these criteria were insufficient to distinguish between fall events and daily activities such as running (Figure 13d), quickly sitting down (Figure 13g), and jumping (Figure 13h).

**Figure 15:**
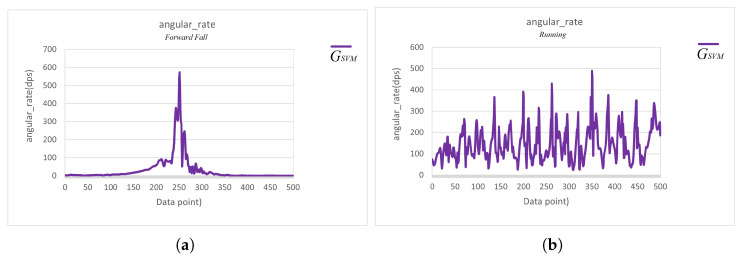
The signal vector magnitude of the gyroscope. (**a**) The signal vector magnitude of the gyroscope during a forward fall. (**b**) The signal vector magnitude of the gyroscope during running activity.

Here, we first explore how to distinguish between running and fall events, and, later, we will further analyze how to identify the two daily activities, quickly sitting down and jumping, that produce $A_{SVM}$ waveforms similar to those of a fall. Further comparison of Figure 12 and Figure 13d reveals that the key to distinguishing fall events from running lay in the waveform changes of the $A_{SVM}$ signal after it exceeded the 1.4 G threshold. As shown in Figure 14, after the $A_{SVM}$ signal exceeded the 1.4 G threshold, the accelerometer’s $A_{SVM}$ signal tended to stabilize during a fall event. In contrast, the signal during running showed continuous, intense fluctuations (as in Figure 13d). Additionally, we compared the gyroscope’s $G_{SVM}$ signal and found that after exceeding the 1.4 G threshold, the $G_{SVM}$ signal corresponding to a fall event also stabilized (as in Figure 15a), while the $G_{SVM}$ signal corresponding to running exhibited continuous fluctuations (as in Figure 15b). These differences served as the key indicators for distinguishing falls from other continuous activities.

Based on the above observations, we calculated the standard deviation of the $A_{SVM}$ and $G_{SVM}$ signals from the 151st to the 200th data point (a total of 50 data points), as in (3) and (4). By calculating the dispersion of the segments, we could distinguish between fall events and running activities [7].

(3) $$\sigma_{\mathrm{Acc}}=\sqrt{\frac{1}{50}\sum_{i=151}^{200}{\left(A_{SVM}\left[i\right]-\mu_{A}\right)}^{2}},$$

where $\mu_{A}$ is the mean value of the $A_{SVM}$ signal from the 151st to the 200th data point.

(4) $$\sigma_{\mathrm{Gyro}}=\sqrt{\frac{1}{50}\sum_{i=151}^{200}{\left(G_{SVM}\left[i\right]-\mu_{G}\right)}^{2}},$$

where $\mu_{G}$ is the mean value of the $G_{SVM}$ signal from the 151st to the 200th data point.

We found that the $\sigma_{\mathrm{Acc}}$ and $\sigma_{\mathrm{Gyro}}$ for the fall samples were much smaller than 100 mg and 10 dps, respectively, while the $\sigma_{\mathrm{Acc}}$ and $\sigma_{\mathrm{Gyro}}$ for non-fall samples were much larger than 100 mg and 10 dps. Therefore, we set 100 mg and 10 dps as the threshold values for $\sigma_{\mathrm{Acc}}$ and $\sigma_{\mathrm{Gyro}}$, respectively. If both conditions in (5) and (6) were satisfied, it was likely that a fall event had occurred. Conversely, if either condition was not met, the action was classified as non-fall daily activity.

(5) $$\sigma_{\mathrm{Acc}}<100\;\mathrm{mg}.$$

(6) $$\sigma_{\mathrm{Gyro}}<10\;\mathrm{dps}.$$

### 3.5. Axis Inclination Angles of the Triaxial Accelerometer

Although the judgment based on the four conditions (four-state machine) could filter out most non-fall activities, there were still a few daily activities that resembled falls and could cause false positives. For example, the quick sitting down in Figure 13g and the jumping motion in Figure 13h. Therefore, we needed additional criteria to improve the accuracy and prevent false positive events.

Among the criteria, we were only using the signal vector magnitude from the accelerometer and gyroscope to differentiate between fall events and daily activities. However, when a fall event occurred, the posture of the person changed, which also led to a change in the posture of the wearable module. As a result, the angle between the module and the horizon (horizontal plane), or the angle between the module and the gravity axis, also changed. Actually, these angles could be calculated based on the *x*, *y*, and *z* components of the accelerometer on the IMU mounted on the device. Therefore, by observing the changes and differences in the angle between the fall detection module and a reference axis during the moment of a fall and various daily activities, we could avoid misclassifying normal daily activities as fall events.

The study in [26] provides a detailed discussion on calculating the inclination angles between different axes and a reference orientation using an accelerometer. In [26], the IMU was placed on a horizontal surface when computing the inclination angles of each axis. However, in this paper, due to the wearing method (as in Figure 10), the circuit board inside the fall detection module was positioned vertically relative to the ground (horizontal plane) during a standing posture. In other words, the IMU chip was also oriented vertically to the horizon. Therefore, the *x*, *y*, and *z* axes defined in this paper differed from those when the IMU chip was in a horizontal orientation. Thus, we had to redefine the inclination angles of each axis and the reference axis accordingly.

In this study, we used the horizontal plane (ground) as the reference and calculated the inclination angles between the IMU’s *x*, *y*, and *z* axes and the horizon, respectively. The symbols for each angle are defined below.

- θ is the angle between the *x*-axis and the horizon.
- ψ is the angle between the *y*-axis and the horizon.
- ϕ is the angle between the *z*-axis and the horizon.

**Figure 16:**
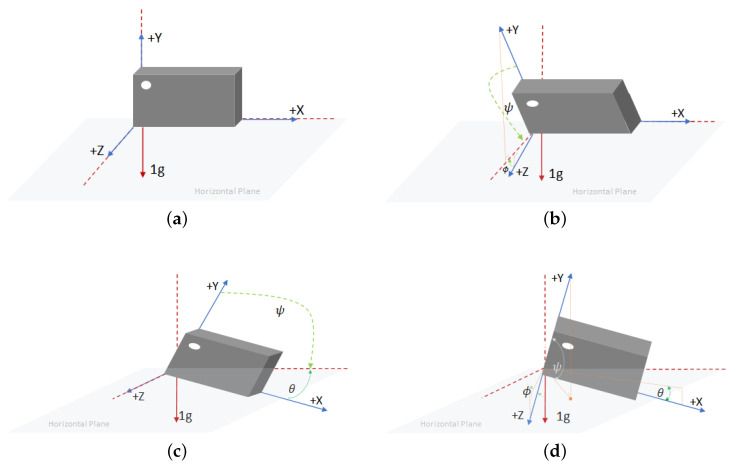
Definition of inclination angles of *x*, *y*, and *z* axes of the IMU sensor (with respect to the horizon). (**a**) Stable standing posture. (**b**) Rotation around the *x*-axis. (**c**) Rotation around the *z*-axis. (**d**) Free rotation.

The three angles θ, ψ, and ϕ could be obtained using (7), (8) and (9), respectively. For better understanding, illustrations of each angle under different IMU postures are shown in Figure 16.

(7) $$\theta =\tan^{-1}\left(\frac{a_{x}}{\sqrt{a_{y}^{2}+a_{z}^{2}}}\right),$$

(8) $$\psi =\tan^{-1}\left(\frac{a_{y}}{\sqrt{a_{z}^{2}+a_{x}^{2}}}\right),$$

(9) $$\phi =\tan^{-1}\left(\frac{a_{z}}{\sqrt{a_{x}^{2}+a_{y}^{2}}}\right),$$

where $a_{x}$, $a_{y}$, and $a_{z}$ are the gravitational acceleration components of the triaxial accelerometer along the *x*, *y*, and *z* axes, respectively.

**Figure 17:**
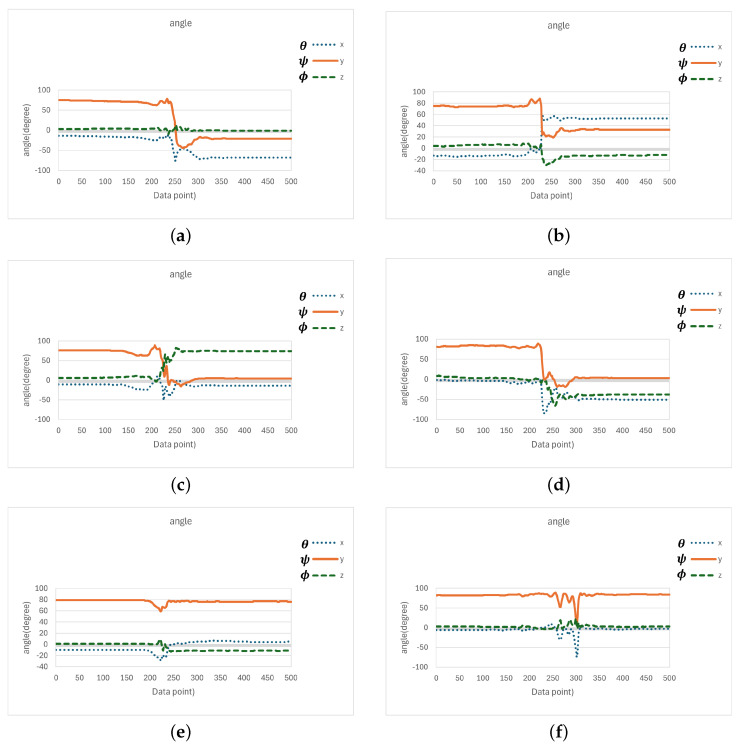
Angle between each axis with respect to the horizontal plane during four types of fall directions and daily activities. (**a**) Forward fall. (**b**) Backward fall. (**c**) Right-side fall. (**d**) Left-side fall. (**e**) Quickly sitting down. (**f**) Jumping.

According to (7)–(9), the angle values (in degrees) of the *x*, *y*, and *z* axes with respect to the horizontal plane were observed and recorded during four fall directions and the two daily activities, quick sitting and jumping, as illustrated in Figure 17. In Figure 17a–d, we can observe that at the moment of a fall event, the angles θ, ψ, and ϕ, representing the angles between the *x*, *y*, and *z* axes and the horizontal plane, could undergo significant changes, depending on the direction of the fall. However, a common observation across all fall directions was that the *y*-axis, which was originally perpendicular to the horizontal plane, rotated as the elderly person fell, causing the angle ψ between the *y*-axis and the horizontal plane to shift from approximately 90 degrees to nearly 0 degrees (due to a 90-degree rotation of the *y*-axis). Moreover, this angular change persisted for a period of time after the fall, until the elderly person either returned to a seated position or stood up.

In contrast, during the daily activities of quick sitting (Figure 17e) and jumping (Figure 17f), although angle changes were also observed, their magnitudes were noticeably smaller compared to those during falls, and the changes were brief, that is, the angles quickly returned to their original values. This difference in angular behavior was therefore highly useful for distinguishing fall events from normal daily activities.

Based on the above observations, during a fall event, the angle ψ between the *y*-axis and the horizontal plane changed from approximately 90 degrees to around 0 degrees. Therefore, we calculated the average ψ angle from the last 20 data points (i.e., the 181st to the 200th point) among the 200 samples collected after the ignition point (i.e., when $A_{SVM}$ was less than 0.8 G), as in (10). If the inequality in (11) was satisfied, i.e., the average ψ value $\bar{\psi }$ was less than 60 degrees, the event was classified as a fall; otherwise, it was considered a normal daily activity.

The reason for selecting the last 20 data points (i.e., the 181st to the 200th) out of the 200 samples collected after the ignition point to calculate the average ψ angle was to avoid interference from the transient phase during the fall process. Typically, a fall could be confirmed within approximately 2 s after the onset of weightlessness (i.e., the ignition point). Therefore, using the 181st to 200th data points helped ensure that the system captured a more stable post-fall state. As for choosing 60 degrees, rather than a smaller angle, as the threshold for classifying ψ, the decision was based on two considerations. First, if the fall detection module was not worn tightly, the ψ angle when the elderly person was lying on the ground could be slightly greater than 0 degrees. Thus, a larger threshold of 60 degrees allowed for greater flexibility in such cases. Second, during normal daily activities such as quick sitting and jumping, the average ψ value typically exceeded 60 degrees. Therefore, setting the threshold at 60 degrees helped reduce the likelihood of false positives. Based on these considerations, this study adopted 60 degrees as the classification threshold. Experimental implementation and validation further confirmed that this threshold effectively distinguished fall events from fall-like daily activities, such as quick sitting and jumping.

(10) $$\bar{\psi }=\frac{1}{20}\sum_{i=181}^{200}\psi \left[i\right].$$

(11) $$\left|\bar{\psi }\right|<60.$$

## 4. Proposed Finite State Machine-Based Fall Detection

Based on the aforementioned step-by-step feature analysis, a fall detection algorithm utilizing a finite state machine and incorporating accelerometer and gyroscope data will be presented in this section. The adoption of a finite state machine (FSM) in the design of the fall detection algorithm in this study was primarily based on two considerations. First, the features identified in the previous section were examined through a step-by-step sequential process, which fit well with the FSM. Second, since the algorithm was intended to be deployed on a wearable device and executed by an MCU, the algorithm’s computational complexity needed to be carefully considered. While it was theoretically possible to wait until all features were collected and then apply a machine learning approach to determine whether a fall had occurred, such an approach would have imposed an excessive computational burden on the MCU, potentially making real-time detection unfeasible.

### 4.1. Threshold Setting Principles for the Finite State Machine

Prior to introducing the proposed finite state machine architecture, we begin by analyzing the process and underlying principles of threshold setting in the FSM. During the development phase of the finite state machine algorithm, we invited five participants to simulate falls and eight types of daily activities. Each participant performed 20 repetitions of each activity. The corresponding IMU data were transmitted via the microcontroller’s built-in Bluetooth Low Energy (BLE) to an offline analysis app for recording and analysis. Additionally, the five participants involved in the development phase did not take part in the final testing phase.

Below, we provide an explanation of how the threshold values were determined for the loss of balance phase, the impact with the ground phase, and the average angle (i.e., $\bar{\psi }$) between the *y*-axis and the horizontal plane after a fall.

#### 4.1.1. $A_{SVM}$ Threshold Decision for the Loss of Balance Phase

**Figure 18:**
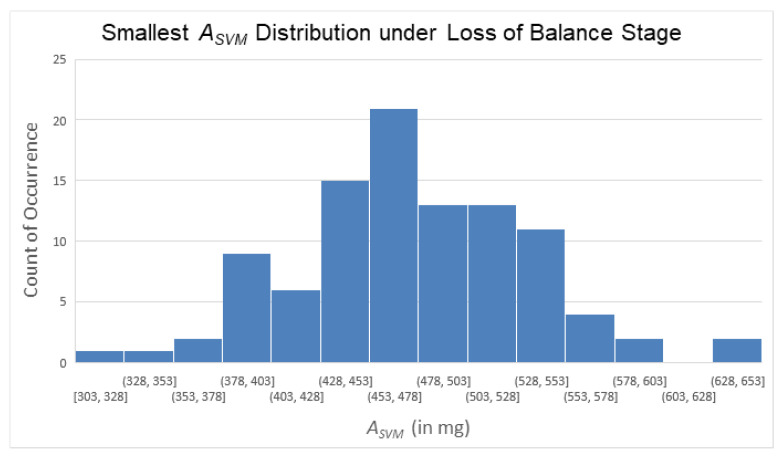
Smallest $A_{SVM}$ distribution under loss of balance stage.

During the loss of balance phase, we collected 100 $A_{SVM}$ signal samples from five participants simulating falls and identified the minimum value during this phase for each sample (i.e., to find the $A_{SVM}$ trough during this stage). The $A_{SVM}$ value distribution corresponding to these troughs is shown in Figure 18. We approximated Figure 18 using a normal distribution; we then calculated the mean (474.56 mg), standard deviation (59.52 mg), and the mean plus three times the standard deviation (653.12 mg) of the distribution. Additionally, we found the largest value among these 100 $A_{SVM}$ troughs, which was 650 mg.

From the above analysis, we observed that $A_{SVM}$ values during this phase of a fall typically dropped below 650 mg. However, to avoid setting an overly strict threshold that might prevent the algorithm from progressing to subsequent decision stages, thus potentially resulting in false negatives, we used a larger integer as the threshold value, and the ignition threshold for the loss of balance phase was set at 800 mg (i.e., 0.8 g). In other words, when the $A_{SVM}$ value fell below 800 mg (i.e., 0.8 g), the subsequent decision process of the FSM was triggered.

#### 4.1.2. $A_{SVM}$ Threshold Decision During Impact with the Ground

**Figure 19:**
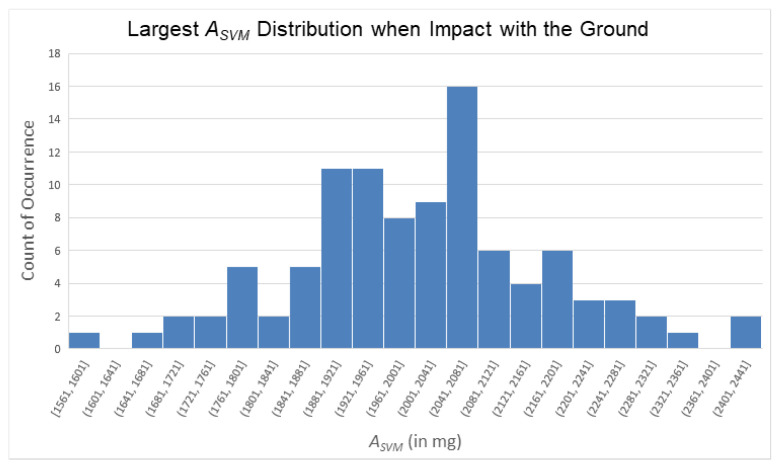
Largest $A_{SVM}$ value distribution during impact with the ground.

Following the same analytical approach used in the loss of balance phase, during the impact with the ground phase, we collected 100 $A_{SVM}$ signals from five participants simulating falls and identified the maximum value for each signal in this phase (i.e., to find the $A_{SVM}$ peak during ground impact). The distribution of these peak values is shown in Figure 19. Similarly, we approximated Figure 19 using a normal distribution and calculated the mean (2007.16 mg), standard deviation (159.48 mg), and the mean minus three times the standard deviation (1528.72 mg) of these 100 peak values. Additionally, we identified the smallest value among these $A_{SVM}$ peaks, which was 1561 mg.

From these 100 data points, we observed that the peak value of $A_{SVM}$ during the ground impact phase reached at least 1561 mg. Considering variations across different trials, and in order to avoid setting an overly strict threshold that might prevent the algorithm from progressing to subsequent decision-making steps, potentially resulting in false negatives, we selected an integer value smaller than the mean minus three times the standard deviation as the threshold. As a result, the threshold for the impact with the ground phase was set at 1400 mg (i.e., 1.4 g). In other words, when the $A_{SVM}$ value reached 1400 mg (i.e., 1.4 g), the subsequent decision process of the FSM was triggered.

#### 4.1.3. Threshold Decision for the Average Angle Between the *Y*-Axis and the Horizontal Plane After a Fall

**Figure 20:**
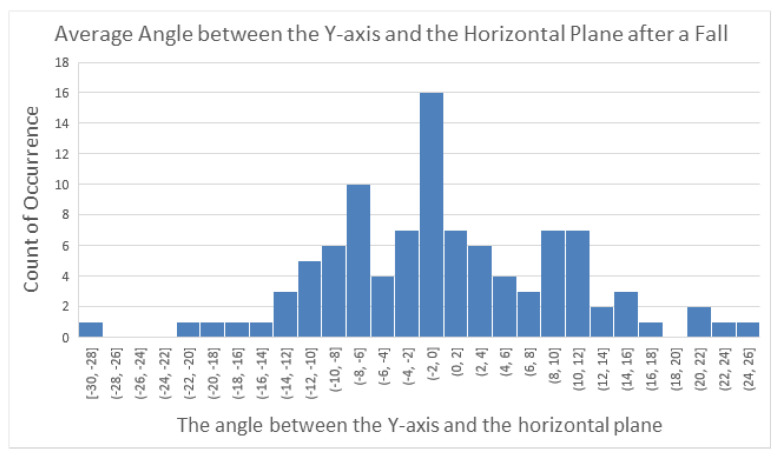
Average angle $\bar{\psi }$ distribution after a fall.

At Stage A3, i.e., after a fall, the angle between the *y*-axis and the horizontal plane tended to approach 0 degrees. To investigate this, we analyzed the last 20 sampled ψ angle values from 100 simulated fall signals collected from 5 participants and calculated their averages, $\bar{\psi }$, to observe the distribution of the angle ψ between the *y*-axis and the horizontal plane after a fall. The distribution of these 100 averaged angle values is shown in Figure 20. Based on this distribution, we found the minimum and maximum values among the 100 averages to be −30 degrees and 25 degrees, respectively.

From this observation, we concluded that after a fall, the angle between the *y*-axis and the horizontal plane typically fell within the range of −30 to +30 degrees. Moreover, during normal physical activities, this angle generally remained around 90 degrees. Considering that sliding or displacement of the module could occur during a fall, we adopted a more relaxed threshold for angle detection. Specifically, when the absolute value of $\bar{\psi }$ was less than 60 degrees, it was considered indicative of a fall event.

### 4.2. The Six-Stage Finite State Machine Architecture

In this study, the proposed fall detection algorithm was categorized into six distinct states, which are described as follows.

1. State 1: This is the initial state, which is responsible for the trigger point check. The MCU monitors whether $A_{SVM}$ falls below 800 mg. If the condition is satisfied, the algorithm transitions to State 2.
2. State 2: This stage aims to evaluate whether a strong reactive force resulting from ground impact is detected following the trigger point. In this stage, 200 samples of tri-axial (*x*, *y*, *z*) data from both the accelerometer and gyroscope are collected, and the corresponding $A_{SVM}$ values are calculated. If any of the 200 samples contain an $A_{SVM}$ value exceeding 1400 mg, the algorithm advances to State 3; otherwise, it returns to State 1.
3. State 3: This stage is intended to evaluate whether the IMU signals are stabilizing. To do so, the standard deviation $\sigma_{\mathrm{Acc}}$ of the last 50 samples (i.e., samples 151 to 200) of the $A_{SVM}$ signal are calculated. If $\sigma_{\mathrm{Acc}}$ is less than 100 mg, the algorithm advances to State 4; otherwise, it returns to State 1.
4. State 4: Similar to State 3, this stage aims to evaluate whether the IMU signals are stabilizing. Specifically, the standard deviation $\sigma_{\mathrm{Gyro}}$ of the last 50 samples (i.e., samples 151 to 200) of the $G_{SVM}$ signal are calculated. If $\sigma_{\mathrm{Gyro}}$ is less than 10 dps, the algorithm advances to State 5; otherwise, it returns to State 1.
5. State 5: This stage aims to evaluate whether the IMU has experienced a change in posture orientation. To do so, the average value $\bar{\psi }$ of the angle ψ (the angle between the *y*-axis and the horizontal plane) is calculated using the last 20 samples (i.e., samples 181 to 200) from the 200 samples taken after the trigger point. If $\bar{\psi }$ is less than 60 degrees, the algorithm advances to State 6; otherwise, it returns to State 1.
6. State 6: The transition to this state indicates that a fall event has been confirmed. At this point, the MCU retrieves the GPS coordinates from the SIM7000G module and transmits the fall event along with the coordinates to the cloud server via NB-IoT. The application on the cloud server then sends an alert message through Line notifications. After completing this action, the system returns to the initial state of the state machine.

**Figure 21:**
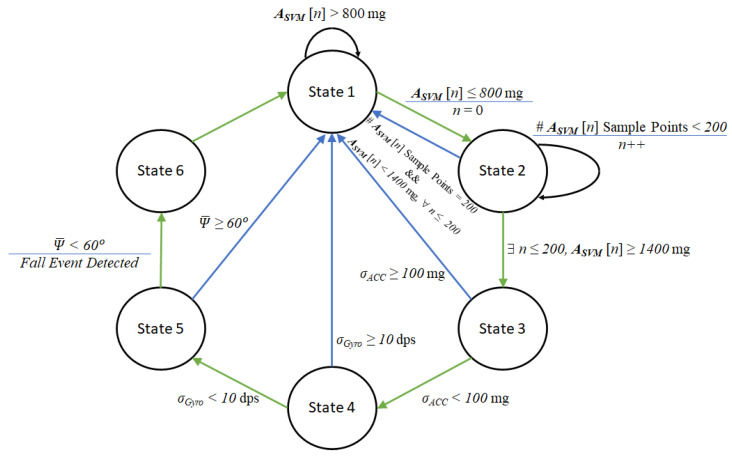
Proposed finite state machine for fall event detection.

The proposed finite state machine for fall event detection is shown in Figure 21. It is worth mentioning that the proposed algorithm incorporates a triggering mechanism (State 1), such that the detection process is only initiated when the signal vector magnitude ($A_{SVM}$) drops below 800 mg (0.8 G). This design helps to avoid unnecessary computation and thus contributes positively to the power efficiency of the wearable device.

## 5. Experimental Results and Comparisons

In this section, we discuss the experimental setup and evaluate the performance of the proposed algorithm. Finally, an analysis of the power consumption is provided to demonstrate the usefulness and effectiveness of the proposed system.

### 5.1. Experimental Scenario

To evaluate the performance of the proposed algorithm, fifteen participants were invited to perform simulated tests involving various activities, including four directions of falls (forward fall, backward fall, right-side fall, and left-side fall) and eight types of daily activities (going upstairs, going downstairs, walking, running, stepping, sitting down, quickly sitting down, and jumping). Each participant performed 50 simulations for each type of fall and 50 simulations for each of the eight daily activities. As a result, each participant contributed a total of 450 data samples (50 fall samples and 400 daily activity samples). All fall simulations were conducted on a yoga mat to ensure safety. Additionally, the test procedures were guided by system-generated instructions to ensure the synchronization of the data timestamps.

Table 1 presents the demographic information of the fifteen participants, including their age, height, weight, and gender. As shown in Table 1, there were variations in age, height, and weight among the participants. This diversity was beneficial for evaluating the effectiveness of the algorithm across different users.

### 5.2. Performance Evaluation Metrics and Results

In this study, we used three metrics, sensitivity (12), specificity (13), and accuracy (14), to evaluate the performance of the system. Each of these metrics had a maximum value of 100%, and higher values indicated better system performance.

(12) $$\mathrm{Sensitivity}=\frac{\mathrm{TP}}{\mathrm{TP}+\mathrm{FN}}$$

(13) $$\mathrm{Specificity}=\frac{\mathrm{TN}}{\mathrm{TN}+\mathrm{FP}}$$

(14) $$\mathrm{Accuracy}=\frac{\mathrm{TP}+\mathrm{TN}}{\mathrm{TP}+\mathrm{TN}+\mathrm{FP}+\mathrm{FN}}$$

In (12), (13), and (14), the terms TP, FP, TN, and FN are used and are defined as follows.

- TP (True Positive): Cases where a fall actually occurred and the system correctly detected it as a fall.
- FP (False Positive): Cases where no fall actually occurred but the system incorrectly detected a fall.
- TN (True Negative): Cases where no fall occurred and the system correctly did not detect a fall.
- FN (False Negative): Cases where a fall actually occurred but the system failed to detect it.

The experimental results are shown in Table 2. The experiments demonstrate that the system achieved an average sensitivity of 97.9%, an average specificity of 99.9%, and an overall average accuracy of 99.7% across the fifteen subjects.

### 5.3. False Positive Analysis and Potential Mitigation Methods

We observed that the system produced five false positive misclassifications, all of which occurred during the action of quickly sitting down. As analyzed in Section 3.5, the $A_{SVM}$ waveform of the triaxial accelerometer during a quick sit-down closely resembled that of a fall event. As a result, the first four states of the finite state machine found it difficult to distinguish between the two actions. The only significant difference between a fall and a quick sit-down lies in the inclination angle ψ between the *y*-axis and the horizontal plane after the action is completed (with ψ close to 0 degrees after a fall and close to 90 degrees after a quick sit-down).

**Figure 22:**
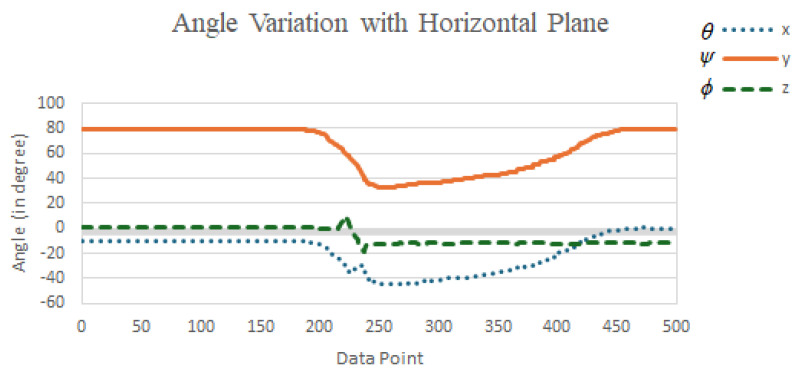
Variation of the angles between the *x*, *y*, and *z* axes and the horizon during a misclassified quickly sitting down activity.

During the evaluation of quickly sitting down, an office chair with a relatively tall backrest was used. In examining these five false positives classified during quick sit-downs, we noticed that the subjects immediately leaned backward after sitting down quickly, leading to a reduced angle between the *y*-axis and the horizontal plane. Figure 22 shows the change in angles between each axis and the horizontal plane for a case where quickly sitting down was misclassified as a fall. From observing the average angle between the *y*-axis and the horizontal plane, we found that due to the soft cushion and tall backrest of the office chair, the subjects tended to lean back after sitting down quickly. Although they subsequently returned to an upright sitting posture (with a more upright torso, rather than a reclined one), this backward-leaning action caused the ψ angle to drop below 60 degrees on average during the last 20 sampling points (i.e., samples 181 to 200 after the $A_{SVM}$ dropped below 0.8g), which led to a false positive classification and a false alarm.

Upon further analysis, we observed that after a fall, the average ψ angle between the *y*-axis and the horizontal plane tended to approach 0 degrees and remained low for an extended period. In contrast, although quickly sitting down could also produce a small ψ angle (due to leaning back), the subject usually returned to an upright posture within a short time, meaning that the ψ angle increased again (as seen in Figure 22). Therefore, to further reduce false positive misclassifications, it may be beneficial to extend the time window used to compute the average ψ angle. For example, instead of averaging over sampling points 181 to 200, we could compute the average over points 181 to 300 or 181 to 400, thereby adding 1 to 2 s of angular observation. This could help exclude false positives caused by quickly sitting down. Obviously, this adjustment would delay the system’s alarm response by 1 to 2 s, depending on the extended observation period. Additionally, the MCU (microcontroller unit) would need to compute and temporarily store the additional angle data during that time.

### 5.4. False Negative Analysis

Besides the five misclassified false positives, we found a total of 16 false negative events that occurred during the 750 fall tests conducted across the fifteen participants, with each subject experiencing similar situations. Upon reviewing these false negative cases, we found that the cause was the device shifting from its original worn position, or even becoming loose or slipping off, due to repeated testing. As a result, the finite state machine only satisfied the conditions of the first four stages, while the final, fifth stage involving the angle determination failed, leading to the system’s inability to detect the fall event.

The reason the device shifted or slipped during repeated tests was that the subjects followed the system’s instructions to perform test actions at fixed time intervals (as described in Section 5.1). Due to limited time, the device’s position was not re-adjusted after each fall test, which occasionally resulted in the device deviating from its original placement or slipping off.

However, these occurrences can actually be regarded as an informal stress test for the system, demonstrating that the device can continue functioning properly even after experiencing strong shaking or impact (such as hitting the ground during a fall). On the other hand, if the device had been properly re-adjusted after every fall test, it is likely that no false negatives would have occurred, and the sensitivity could have reached 100%. Through these experiments, it was also confirmed that the finite state machine algorithm, comprising the six defined states, was effective in resisting interference from daily activities and maintained high sensitivity in detecting fall events.

### 5.5. Power Consumption Analysis

Wearable devices typically prioritize lightweight design and thus are often powered by relatively small-capacity lithium-ion batteries. As a result, the operating power consumption of the device becomes critically important. In this study, a 2000 mAh lithium battery was used as the power source. To evaluate the power consumption of the proposed device, we conducted two types of endurance tests and performed a theoretical estimation. Endurance Test A involved activating all functionalities of the device. In this scenario, the SIM7000G module, specifically its GNSS and NB-IoT components, constituted the primary power-consuming elements. The GNSS was configured to continuously track positioning data, while the NB-IoT remained in the RRC Connected state. Additionally, the microcontroller unit (MCU) operated at full speed (64 MHz) throughout the test. This configuration was designed to assess the device’s maximum power consumption and operational time under worst-case conditions. Endurance Test B focused on a more power-efficient scenario. In this test, the SIM7000G module was set to Normal Idle mode, in which the GNSS was disabled (Wake-up on demand) and the NB-IoT module remained in the RRC Idle state. The MCU still operated at full speed (64 MHz). This setup aimed to simulate a more typical usage condition and measure corresponding improvements in power efficiency. Theoretical estimation was conducted as the third approach. In this scenario, the MCU was placed in a low-power sleep mode, and only awakened via an interrupt triggered by the IMU’s “Wake-on-Shake” function. Based on specifications provided in the component datasheets, we conducted a theoretical analysis to estimate the device’s average current consumption under the optimized configuration. During all endurance tests, current measurements were carried out using the GDM-8261A, a 6½-digit digital multimeter. Detailed explanations of each test and analysis scenario are provided below.

**Endurance** **Test A.** Baseline Power Consumption and Battery Endurance Test (All Functions Enabled):
  The proposed system integrated the following components:
  - Microcontroller Unit STM32WB5MM: Operating in Run Mode at 64 MHz.
  - Inertial Measurement Unit LSM6DSO: Operating in continuous mode.
  - GNSS and NB-IoT Module SIM7000G: Operating in standby mode, with GNSS enabled (Continuous Tracking) and NB-IoT in RRC Connected state.
  - Power Management IC TPS631010: For stable buck-boost voltage regulation.
  Under this configuration, the average current consumption of the device was approximately 72 mA. The duration of the test was 10 h, and the battery voltage dropped from a fully charged 4.2 V to approximately 3.8 V. Additionally, the remaining battery capacity could be calculated based on the initial capacity and average discharge current, as shown in (15).
  (15) $$C_{rem}=C_{init}-\left(I_{dis}\times t\right),$$
  where $C_{rem}$ is the remaining capacity (in mAh), $C_{init}$ is the initial capacity (in mAh), $I_{dis}$ is the discharge current (in mA), and *t* is the discharge time (in hours). In this study, the initial battery capacity was 2000 mAh, with a discharge current of 72 mA over a discharge time of 10 h. Therefore, the remaining capacity was calculated as (2000 mAh-72 mA × 10 h) = 1280 mAh, which corresponded to $\frac{1280}{2000}\times 100\%$ = 64% of the original battery capacity. Moreover, if the device was operated continuously until the battery was depleted, the operating time of the 2000 mAh lithium-ion battery was calculated as follows:
  $$\mathrm{Operating}\;\mathrm{time}=\frac{2000\;\mathrm{mAh}}{72\;\mathrm{mA}}\approx 27.8h$$
  This indicated that the system could operate continuously for over one day under the most active condition (all functions enabled).
**Endurance** **Test B.** Power-Saving Mode Endurance Test:
  We also analyzed the impact of lower power consumption modes on the system’s operational duration. To this end, we conducted an additional endurance test under a power-saving configuration.
  - Microcontroller Unit STM32WB5MM: Operating in Run Mode at 64 MHz.
  - Inertial Measurement Unit LSM6DSO: Operating in continuous mode.
  - SIM7000G configured to Normal Idle mode with GNSS disabled (wake-up on demand) and NB-IoT in the RRC Idle state.
  Under this condition, the average current consumption was reduced to approximately 20 mA, resulting in
  $$\mathrm{Operating}\;\mathrm{time}=\frac{2000\;\mathrm{mAh}}{20\;\mathrm{mA}}=100\;h\approx 4.2\;\mathrm{days}$$
**Theoretical** **Estimation C.** Ultra-Low Power Mode (Estimated):
  If a further reduction in power consumption is required, the microcontroller can be configured to operate in a low-power mode and wake only when necessary, for example, by utilizing the IMU’s “Wake-on-Shake” feature. This approach can further extend the device’s operational time. The corresponding configurations are as follows:
  - The LSM6DSO IMU configured in "Wake-on-Shake" mode.
  - STM32WB5MM in STOP or STANDBY low-power mode, woken via interrupt upon motion.
  - GNSS and NB-IoT activated only when needed for location acquisition and data transmission.
  Under this condition, the estimated average current is 5 mA, and the corresponding operating time is shown below.
  $$\mathrm{Operating}\;\mathrm{time}=\frac{2000\;\mathrm{mAh}}{5\;\mathrm{mA}}=400\;h\approx 16.7\;\mathrm{days}$$

With the above endurance tests and theoretical analysis, it can be confirmed that not only does the proposed device demonstrate excellent sensitivity and specificity but its low power consumption also meets the requirements for long-term wear, ensuring comprehensive protection of the wearer’s personal safety.

### 5.6. Comparisons to Existing State-of-the-Art Fall Detectors

Next, we compare the algorithm proposed in this paper with three state-of-the-art wearable fall detection algorithms, including those in [6,8,27]. The comparison between the proposed approach and the aforementioned references is presented in Table 3, while the types of activities tested by each algorithm are detailed in Table 4.

In [6], the wearable module integrated an accelerometer, gyroscope, and pressure sensor, utilizing spectral features and a feedforward neural network (FFNN) to classify falls and daily activities. To conserve energy, the algorithm was activated only when the measured acceleration exceeded a threshold of 2.5 g. This approach achieved high accuracy and low latency, making it well-suited for real-time detection. Similar to the algorithm proposed in this paper, ref. [6] employed a trigger mechanism to reduce overall power consumption. However, it extracted 42 frequency–domain features from a 10-second window as input to the model, which required performing FFT during inference, potentially imposing computational and power burdens on the microcontrollers. Additionally, the system described in [6] did not provide positioning functionality, and its fall event notifications relied on GSM communication. Since the GSM service has been discontinued in most developed countries (e.g., the United States, Canada, Japan, South Korea, Australia, Singapore, and Taiwan), the effectiveness of its emergency alert functionality is significantly limited.

The study in [8] presented a fall detection system based on wearable glasses, employing an IMU embedded in the glasses frame, along with threshold-based detection to identify fall events. The study involved five participants and tested three types of falls—trip falls, slip falls, and lateral falls—as well as five types of daily activities, including running, walking, jumping, sitting down and standing up, and bending down. Reported sensitivity, specificity, and accuracy reached 95.83%, 97.33%, and 95.44%, respectively. Although [8] also utilized an IMU, the daily activities tested differ from those in this work. To ensure comparability, additional simulations were conducted for the sit down and stand up and bend down activities mentioned in [8]. Five participants performed each activity 10 times, with no false positives recorded. Notably, the study in [8] did not include high false-positive-prone activities such as quickly sitting down or jumping, which were considered in this study. Therefore, the specificity achieved in this work is demonstrably higher than that in [8]. Regarding fall event notifications, the study in [8] used Wi-Fi connectivity to send alerts via email and Short Message Service (SMS). As a result, its communication capabilities are limited when Wi-Fi access is unavailable.

In [27], a wearable fall detection system combining an IMU and a barometric altimeter was presented. It used a two-stage algorithm: the first stage detected fast falls by thresholding downward vertical velocity (≥1.38 m/s), enabling detection before impact (157 ms early); the second stage confirmed slower falls using features such as impact, height change, and posture. According to [27], the system could achieve 100% sensitivity, specificity, and accuracy under certain configurations. However, while the study simulated various daily activities, it did not include boundary scenarios like quickly sitting down or jumping, which are known to cause false positives. Additionally, the fall detection computation was not performed on the wearable device itself but instead involved data transmission from the MCU via Bluetooth to a smartphone, which then uploaded the data to a laptop over Wi-Fi for offline processing in MATLAB. This architecture makes the system dependent on both a mobile device and a wireless connection, and thus it is not suitable for real-time detection. Finally, the system lacks positioning functionality and fall alert mechanisms, making it unsuitable for outdoor use or enabling immediate emergency response.

Compared to the referenced works, this study employed a low-complexity finite state machine algorithm, achieving a sensitivity of 93.6%, specificity of 99.9%, and accuracy of 99.19%. All computations were performed on the device’s MCU. Upon a fall event, the system could obtain the GPS coordinates of the incident location via the GPS receiver integrated into the SIM7000G module. The coordinates and alert message were then transmitted to a cloud server via NB-IoT and subsequently pushed to designated recipients through an instant messaging application along with a map link. Therefore, the proposed system not only features low complexity and real-time processing capability but also offers low power consumption and built-in positioning functionality, making it suitable for long-term wear and outdoor use. It ensures that elderly users can receive timely assistance immediately after an accident occurs.

## 6. Limitations and Future Work

In this section, we describe scenarios in which the proposed system may encounter GNSS (specifically GPS) positioning failures. We also explore alternative positioning methods that can be employed when GNSS signals are unavailable. Furthermore, given the rapid advancements in machine learning and artificial intelligence, this section also investigates the potential of integrating these technologies into microcontroller-based fall detection algorithms. Particular attention is given to the constraints of wearable devices, which require a compact form factor, low computational complexity, and long-term usability. The ultimate goal is to facilitate the development of next-generation, user-customized fall detection systems, in which algorithm parameters can be adaptively optimized based on individual users.

### 6.1. GPS Limitations and Positioning Alternatives Without GPS Signals

In our current study, GNSS (specifically GPS) was primarily employed to enhance the safety of elderly individuals during outdoor activities. If a fall occurs in open environments, such as parks, sidewalks, or public spaces, GPS enables immediate location reporting, allowing for timely assistance from caregivers or emergency responders. Ensuring outdoor safety is a central objective of the system at this stage. However, GPS is not reliable in indoor scenarios, such as residential buildings or malls, where signal obstruction often prevents accurate positioning. Therefore, enabling effective indoor localization is essential for future system enhancements. Below, we outline several possible positioning methods that can be used when GPS signals are unavailable.

- Wi-Fi-based positioning:
  Utilizing a received signal strength indicator (RSSI) or fingerprinting from nearby access points to estimate indoor location with room-level accuracy, leveraging existing Wi-Fi infrastructure. This approach requires either replacing the current module with one that supports Wi-Fi functionality or adding a separate Wi-Fi chip to the existing module.
- Inertial Navigation System (INS):
  Using accelerometers and gyroscopes to estimate short-term motion and location via dead reckoning. While INS alone is prone to drift, it can be combined with other methods for improved accuracy.
- Bluetooth Low Energy (BLE)-based positioning:
  Our current hardware platform, the STM32WB5MMG microcontroller, already integrates BLE capability. This enables the future integration of BLE-based indoor localization using methods such as trilateration from fixed BLE beacons or proximity-based detection. BLE positioning offers a cost-effective, energy-efficient solution suitable for indoor environments like smart homes or eldercare facilities.
- Ultra-Wideband (UWB):
  Although not currently implemented, UWB offers superior accuracy (within 10–30 cm) and may be explored in future system versions for high-precision tracking in specific use cases. This approach requires adding a UWB transceiver chip, such as the Qorvo DW1000, to the existing module.
- Sensor fusion:
  Combining the above techniques (e.g., BLE + INS or Wi-Fi + INS) through filtering algorithms like Kalman filters can mitigate individual limitations and provide robust indoor tracking performance.

By leveraging the BLE capability already embedded in our system’s MCU, we believe that indoor positioning functionalities can be practically added with minimal hardware modification. This expands the system’s applicability beyond outdoor environments, supporting a more comprehensive fall localization solution.

### 6.2. Integrating Artificial Intelligence into Next-Generation User-Customized Devices

The proposed fall detection system is implemented using a finite state machine (FSM) on a microcontroller platform to ensure real-time operation, low power consumption, and extended wearable usage time. While this design achieves high efficiency and accuracy, it also introduces certain limitations and areas for future exploration.

First, the FSM architecture, while computationally lightweight, lacks the adaptability of learning-based models. Its decision process relies on a set of manually defined thresholds for fall-related features, which may not be optimal across diverse user profiles. Variations in individual behaviors, such as rapid sitting or atypical gait patterns, can lead to false positives or false negatives if fixed thresholds are used.

Second, due to hardware constraints of the microcontroller, complex machine learning models are not suitable for real-time onboard execution. Therefore, personalization of the FSM parameters must be performed through efficient offline methods.

To address these limitations, future work will focus on developing user-specific threshold optimization techniques. Before deployment, users may be asked to record brief segments of daily activity data (e.g., walking, sitting), which can serve as a calibration set. Based on this data, several artificial intelligence (AI)-driven approaches will be explored to personalize FSM parameters without compromising computational efficiency. Potential directions include the following:

A. Offline Calibration Using Individual Activity Data:
  Before deploying the device, brief data collection from the user’s typical daily activities, especially those prone to being misclassified (e.g., quick sitting), can be used for parameter refinement.
B. AI-Driven Parameter Optimization (Offline):
  Although full deep learning is unsuitable for microcontroller execution, AI models can be trained offline to suggest or adapt FSM thresholds. Several strategies will be explored:
  - Meta-learning approaches (e.g., MAML, Reptile) for few-shot personalization using a small amount of user data.
  - Neural networks for parameter recommendation, mapping user profiles or calibration patterns to threshold values.
  - Bayesian optimization to efficiently search for optimal threshold combinations, minimizing misclassification.
  - Clustering-based transfer learning, where users are grouped by movement similarity and share optimized parameter sets.
C. Low-Complexity Machine Learning Alternatives:
  Replacing certain FSM states with low-complexity classifiers such as linear–kernel support vector machines (SVMs) could improve adaptability while retaining microcontroller compatibility. These models can be optimized offline to ensure lightweight deployment.
D. Reinforcement Learning for Adaptive Tuning:
  In longer-term development, reinforcement learning techniques may be applied to continuously refine thresholds during use, based on system feedback and performance metrics.

Overall, these future enhancements aim to integrate the efficiency of FSMs with the adaptability of AI, resulting in systems that are not only energy-efficient and capable of real-time processing but also tailored to individual user characteristics, paving the way for the next generation of personalized, lightweight, and intelligent fall detection devices.

## 7. Conclusions

This paper presented a wearable fall detection system based on an accelerometer and gyroscope, designed to provide a real-time detection, localization, and notification solution for fall incidents that may occur among elderly individuals in outdoor environments. Considering the requirements for lightweight design and low power consumption in wearable devices, we employed a low-complexity finite state machine algorithm for fall detection, with all computations performed on the module’s MCU. The experimental results demonstrate excellent performance, with an average sensitivity of 97.9%, specificity of 99.9%, and accuracy of 99.7%, based on data collected from fifteen participants performing 400 samples of daily activities and 50 fall samples each. Upon detecting a fall, the system retrieves the current GPS coordinates via the SIM7000G module’s built-in GPS receiver. The coordinate, along with an alert message, are transmitted to a cloud server via NB-IoT. The server then pushes the location map and alert notification to family members or designated contacts through an instant messaging application. For power consumption evaluation, a 10 h continuous test was conducted with all primary functions active. After 10 h of operation, approximately 64% of the battery capacity remained, confirming the system’s suitability for long-term wear. The implementation of this device is expected to significantly enhance safety for elderly individuals during outdoor activities, ensuring timely assistance in the event of an accident, while also reducing the burden on families and the overall cost of elderly care to society.

## Figures and Tables

**Table 1:** Participant information.

	Age	Height	Weight	Gender
Participant01	27	163 cm	49 kg	Female
Participant02	48	165 cm	60 kg	Female
Participant03	53	168 cm	57 kg	Female
Participant04	68	150 cm	46 kg	Female
Participant05	22	190 cm	55 kg	Female
Participant06	26	172 cm	70 kg	Male
Participant07	26	173 cm	85 kg	Male
Participant08	26	171 cm	78 kg	Male
Participant09	27	183 cm	60 kg	Male
Participant10	35	173 cm	85 kg	Male
Participant11	38	178 cm	70 kg	Male
Participant12	43	177 cm	90 kg	Male
Participant13	51	172 cm	85 kg	Male
Participant14	57	180 cm	82 kg	Male
Participant15	60	179 cm	75 kg	Male
Average	40.47	172.93 cm	70.67 kg	-
SD	14.91	9.41 cm	15.75 kg	-

**Table 2:** Performance of the proposed finite state machine-based fall detection algorithm.

Activities	Fall Down	Going Upstairs	Going Downstairs	Walking	Running	Stepping	Sitting Down	Quickly Sitting Down	Jumping
Samples	750	750	750	750	750	750	750	750	750
True Positive	734	-	-	-	-	-	-	-	-
False Positive	-	0	0	0	0	0	0	5	0
True Negative	-	750	750	750	750	750	750	745	750
False Negative	16	-	-	-	-	-	-	-	-
Sensitivity	0.979								
Specificity	0.999								
Accuracy	0.997								

**Table 3:** Comparison with state-of-the-art fall detection algorithms.

Item	Proposed	[6]	[8]	[27]
Processing Unit	MCU STM32WB5MMG	MCU STM32U575xx	Arduino MCU	NXP LPC1768 Smartphone Samsung Galaxy SII
Device Location	Waist	Waist	Head	Waist
Algorithm	FSM	Deep Learning- based Algorithm	FSM	Threshold- based Algorithm
Test Data Count	Fall 50 Non-Fall 400	Fall 225 Non-Fall 437	Fall 120 Non-Fall 450	Fall 375 Non-Fall 3 hours
Sensors Used	Accelerometer Gyroscope	Accelerometer Gyroscope	Barometer	Accelerometer Gyroscope Barometer
Accuracy (%)	99.7%	99.38%	95.83%	100%
Sensitivity (%)	97.9%	99.79%	97.33%	100%
Specificity (%)	99.9%	98.62%	95.44%	100%
Alarm Notification	Line	GSM	E-mail +SMS	N/A
Communication Method	NB-IoT	GSM	WiFi	Bluetooth + WiFi For offline analysis
Automatic Connection	O	O	X	O

**Table 4:** Test activities of the proposed approach and those of comparison algorithms.

Activity	Proposed	[6]	[8]	[27]
Going upstairs	✓	✓	✓	✓
Going downstairs	✓	✓	✓	✓
Walking	✓	✓	✓	✓
Running	✓	✓	✓	✓
Stepping	✓	✓	✓	✓
Sitting down	✓	✓	✓	✗
Sitting down quickly	✓	✓	✓	✓
Jumping	✓	✗	✗	✗
Bending down	✓	✗	✗	✓
Standing up	✓	✗	✗	✓
Turning while walking	✓	✗	✗	✓

## Data Availability

The data presented in this study are openly available at https://github.com/jasonkau/fall-detection-dataset-IMU (last accessed on 9 June 2025).

## Abbreviations

The following abbreviations are used in this manuscript:

GNSS	Global Navigation Satellite System
GPS	Global Positioning System
NB-IoT	Narrowband Internet of Things
IMU	Inertial Measurement Unit
MCU	Microcontroller Unit
KNN	K-Nearest Neighbor
SVM	Support Vector Machine
CNN	Convolutional Neural Network
RNN	Recurrent Neural Network
APP	Application Program
BLE	Bluetooth Low Energy
PCB	Printed Circuit Board
LTE	Long-Term Evolution
SVM	Signal Vector Magnitude
SMS	Short Message Service
